# Resting-State Functional Connectivity Between the Cingulo-Opercular and Default Mode Networks May Explain Socioeconomic Inequalities in Cognitive Development

**DOI:** 10.31586/jcn.2025.1241

**Published:** 2025-02-25

**Authors:** Shervin Assari, Alexandra Donovan, Golnoush Akhlaghipour, Mario F Mendez

**Affiliations:** 1 Department of Internal Medicine, Charles R. Drew University of Medicine and Science, Los Angeles, CA, United States; 2 Department of Family Medicine, Charles R. Drew University of Medicine and Science, Los Angeles, CA, United States; 3 Department of Urban Public Health, Charles R. Drew University of Medicine and Science, Los Angeles, CA, United States; 4 Marginalization-Related Diminished Returns (MDRs) Center, Los Angeles, CA, United States; 5 Department of Neurology, University of California Los Angeles (UCLA), Los Angeles, CA, USA; 6 Department of Psychiatry & Biobehavioral Sciences, University of California Los Angeles (UCLA), Los Angeles, CA, USA

**Keywords:** Cingulo-Opercular Network, Default Mode Network, Cognition, Educational Opportunities, Family Structure, Resting-State Functional Connectivity, Social Determinants of Health

## Abstract

**Background:**

The Cingulo-Opercular Network (CON) is a crucial executive control network involved in regulating actions and facilitating higher-order cognitive processes. Resting-state functional connectivity between the CON and the Default Mode Network (DMN) plays a vital role in cognitive regulation, enabling the transition between internally focused and externally directed tasks. This study investigates whether resting-state functional connectivity between the CON and DMN mediates the effects of social determinants, such as educational opportunities and family structure, on cognitive outcomes in youth.

**Aims:**

This study aims to explore how CON-DMN connectivity influences the relationship between social gradients and cognition in youth. Specifically, it examines whether resting-state functional connectivity between these networks mediates the effects of educational opportunities and family structure on cognitive outcomes and seeks to uncover the neural mechanisms underlying these social gradients.

**Methods:**

Data were derived from the Adolescent Brain Cognitive Development (ABCD) study, a large longitudinal dataset of over 11,000 children aged 9–10 years. Cognitive outcomes were assessed using standardized NIH toolbox measures: Total Composite, Fluid Reasoning, Picture Vocabulary, Pattern Recognition, and Card Sorting. Social determinants were operationalized using indicators such as parental education, family composition, and neighborhood educational opportunities (COI). Resting-state functional connectivity (rsFC) between the CON and DMN was measured using functional magnetic resonance imaging (fMRI). Structural equation modeling (SEM) was employed to test whether CON-DMN rsFC mediated the relationship between social determinants and cognitive outcomes, adjusting for potential confounders such as age, sex, and race/ethnicity.

**Results:**

Stable family structure and greater educational opportunities were significantly associated with improved cognitive performance. These relationships were mediated by reduced functional connectivity between the CON and DMN.

**Conclusion:**

Reduced functional connectivity between the CON and DMN serves as a neural mechanism linking social gradients, such as educational opportunities and family structure, to better cognitive outcomes in youth.

## 1. Introduction

Cognitive development during childhood is profoundly influenced by social determinants [1–4]. Children from high socioeconomic status (SES) backgrounds are more likely to attend well-resourced schools, providing them with enriched learning environments [5–7]. Conversely, children raised in poverty often receive less cognitive stimulation, which hinders their developmental trajectories [8–10].

Neural pathways, including specific brain regions, structures, networks, and the functional connectivity between them, predict cognitive function [11–13]. Among the essential networks involved in cognitive processes is the Cingulo-Opercular Network (CON) [14–16], which plays a pivotal role in executive control and goal-directed behaviors. The CON regulates actions through top-down control mechanisms, facilitating higher-order cognitive functions such as attention, error detection, and task maintenance. Similarly, the Default Mode Network (DMN) is crucial for cognitive processes, primarily active during rest and self-referential thought, and deactivating during externally focused, task-oriented activities. Functional connectivity between these networks—the CON and DMN—has emerged as a key neural substrate for cognitive regulation, enabling smooth transitions between introspective and goal-directed states [16–19].

Social determinants, such as educational opportunities and family structure, also significantly shape the neural pathways that underlie cognitive outcomes during formative years [20–25]. Educational opportunities, often represented by parental education and neighborhood socioeconomic status (SES), provide cognitive stimulation and an enriched environment that supports brain maturation and cognitive growth [26–31]. Family structure, including marital status and household composition, contributes to stability and emotional support, further shaping developmental trajectories. However, disparities in these social determinants contribute to social gradients in cognition, where children from less advantaged backgrounds consistently exhibit poorer cognitive outcomes compared to their more privileged peers.

While the influence of social determinants on cognitive and brain development is well-established [30,32–35], there is a need to identify the neural mechanisms that mediate these relationships. Recent advancements in neuroimaging, particularly resting-state functional connectivity (rsFC), provide a robust framework for examining how social factors influence neural network connectivity and, consequently, cognitive performance. rsFC between the CON and DMN is known to support cognitive flexibility and executive function [18,36,37], and is shaped by social factors such as SES [38]. These attributes are critical for academic success and adaptive behaviors, suggesting that CON-DMN connectivity may serve as a mediating mechanism through which social opportunities and stability influence cognitive function.

This study utilizes data from the Adolescent Brain Cognitive Development (ABCD) study [39–44], a large longitudinal cohort of children aged 9–10 years, to explore whether functional connectivity between the CON and DMN mediates the relationship between social determinants—specifically educational opportunities and family structure—and cognitive outcomes. By integrating measures of social context, neural connectivity, and cognitive performance, this research aims to clarify the pathways through which social gradients in cognition are established and perpetuated. Understanding these mechanisms is essential for designing interventions to address disparities in cognitive development and promote equity in educational and developmental outcomes.

## 2. Methods

### 2.1. Study Design and Participants

This study utilized data from the Adolescent Brain Cognitive Development (ABCD) Study, a longitudinal, multisite cohort study designed to explore factors influencing brain development and health in youth across the United States. We analyzed baseline data collected from a diverse sample of nearly 12,000 children aged 9–10 years, focusing on demographic, social, and neuroimaging variables. The study population included children from a range of racial/ethnic backgrounds and socioeconomic contexts.

### 2.2. Measures

#### 2.2.1. Demographic and Social Variables

Key demographic variables included age (in years), sex (male vs. female), and race/ethnicity (categorized as Black, Latino, Asian, Other, with White as the reference group). Socioeconomic indicators comprised parental years of education, household marital structure (married vs. not married), and the Child Opportunity Index (COI) Educational Subscale (national level), which reflects regional educational opportunities.

#### 2.2.2. Resting-State Functional Connectivity (rsFC)

rsFC between the Cingulo-Opercular Network (CON) and Default Mode Network (DMN) were drawn from ABCD data set. The ABCD team have calculated the rsFC using functional magnetic resonance imaging (fMRI). Standard preprocessing pipelines, including motion correction, spatial normalization, and filtering, were applied to derive connectivity metrics. CON-DMN rsFC was quantified as the Fisher’s z-transformed correlation between mean time series of the two networks.

### 2.3. Harmonization

The MRI procedures used in the ABCD Study are thoroughly explained in detail in other publications [39–44]. The ABCD Imaging Acquisition Workgroup (https://abcdstudy.org/scientists-workgroups.html) developed, refined, and standardized the imaging measures and protocols across all 21 ABCD sites. This referenced work outlines the foundation and methodology of the ABCD imaging protocols and provides an initial assessment of their quality, demonstrating their suitability for children aged 9 to 10 years [45].

#### 2.3.1. Cognitive Outcomes

Cognitive performance was measured using the following measures from the NIH cognitive toolbox [46–48]: Reading, Total Composite, Fluid Reasoning, Picture Vocabulary, Pattern Recognition, and Card Sorting. These tasks capture a range of cognitive domains relevant to academic and everyday functioning. For this analysis, we used a latent factor for cognitive function.

### 2.4. Statistical Analysis

Structural equation modeling (SEM) was employed to examine the relationships between demographic and social variables, rsFC, and cognitive outcomes. Two models were developed: one predicting rsFC from demographic and social variables, and the other predicting cognitive outcomes from rsFC and social factors. Model coefficients (B), standard errors (SE), and p-values were reported for all paths. Age and sex were included as covariates in all models. The SEM allowed both direct effects from socioeconomic indicators to cognitive function as well as indirect effects from socioeconomic indicators to cognitive functions via rsFC between DMN and CON. Model fit was assessed using the Comparative Fit Index (CFI), Tucker-Lewis Index (TLI), and Root Mean Square Error of Approximation (RMSEA) [49]. A CFI and TLI above 0.90 and an RMSEA below 0.08 indicated acceptable model fit. Missing data were handled using full information maximum likelihood estimation. All analyses were conducted using Stata 18.0.

### 2.5. Ethical Considerations

The ABCD Study protocol was approved by the institutional review board of the UCSD. Informed consent and assent were obtained from parents and children, respectively. Data were analyzed anonymously.

## 3. Results

**Figure 1.**
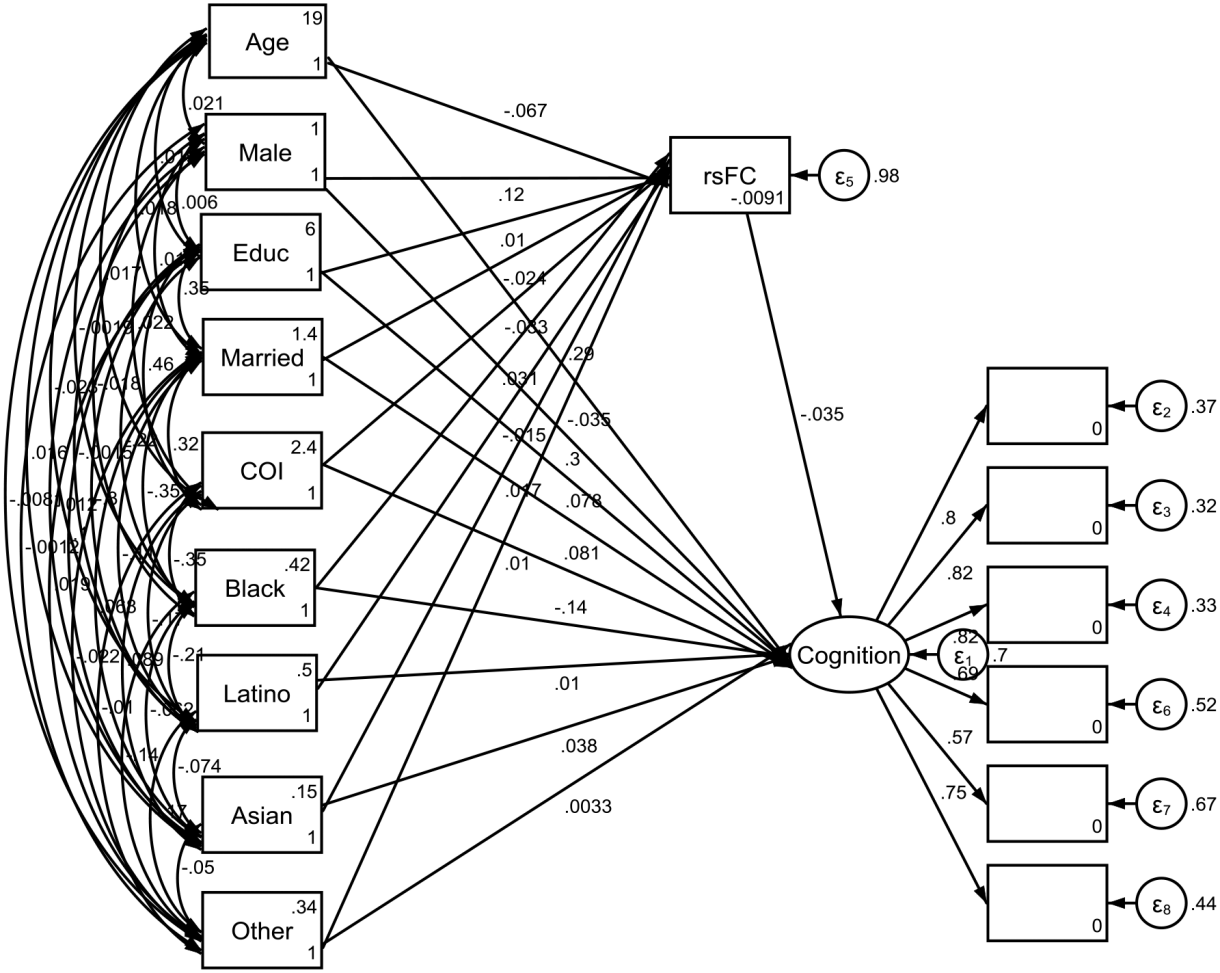
Summary of Structural Equation Model (SEM) Note: Cognitive Function Observed Variables Include 1) Reading, 2) Total Composite, 3) Fluid, 4) Picture, 5) Pattern, and 6) Card Sortl Educ: Parental Education; Married: Parents Married; COI: Childhood Opportunity Index

As shown in Table 1 and Figure 1, our structural equation modeling (SEM) revealed significant relationships between demographic and social variables, resting-state functional connectivity (rsFC) between the Cingulo-Opercular Network (CON) and the Default Mode Network (DMN), and cognitive outcomes. These findings are detailed below:

### 3.1. Associations with rsFC

Living in a married household was associated with reduced rsFC (B = −0.024, SE = 0.011, p = 0.034). Educational opportunities (COI Educational National) were negatively associated with rsFC, indicating that children from regions with higher educational opportunities had lower CON-DMN connectivity (B = −0.033, SE = 0.012, p = 0.007). Parental education years showed no significant association with rsFC (B = 0.010, SE = 0.012, p = 0.403). Race/ethnicity influenced rsFC. Black children had significantly higher rsFC compared to the reference group (B = 0.031, SE = 0.012, p = 0.009). Latino, Asian, and Other racial/ethnic groups did not show significant differences from the reference group. Age was negatively associated with rsFC, indicating that older children demonstrated lower connectivity between the CON and DMN (B = −0.067, SE = 0.010, p < 0.001). Male sex was positively associated with rsFC, with boys showing higher functional connectivity (B = 0.123, SE = 0.010, p < 0.001).

### 3.2. Associations with Cognition

rsFC was negatively associated with cognition, with lower CON-DMN connectivity predicting better cognitive performance (B = −0.035, SE = 0.009, p < 0.001). Living in a married household and educational opportunities were positively associated with cognitive outcomes (Married household: B = 0.078, SE = 0.009, p < 0.001; Educational opportunities: B = 0.081, SE = 0.011, p < 0.001). Race/ethnicity effects varied. Black children had significantly lower cognitive outcomes compared to the reference group (B = −0.139, SE = 0.010, p < 0.001), while Asian children showed a small positive association (B = 0.038, SE = 0.008, p < 0.001). Latino and Other racial/ethnic groups showed no significant differences. Age and parental education years were strong positive predictors of cognitive outcomes (Age: B = 0.295, SE = 0.005, p < 0.001; Parental education: B = 0.299, SE = 0.010, p < 0.001). Male sex was negatively associated with cognition (B = −0.035, SE = 0.008, p < 0.001).

### 3.3. Cognitive Outcomes by Task

The latent factor of Cognition demonstrated strong loading of items of cognitive ability using these task-specific measures: Reading (B = 0.797, SE = 0.003, p < 0.001), Total Composite (B = 0.823, SE = 0.004, p < 0.001), Fluid reasoning (B = 0.817, SE = 0.004, p < 0.001), Picture Vocabulary (B = 0.692, SE = 0.004, p < 0.001), Pattern Recognition (B = 0.573, SE = 0.004, p < 0.001), and Card Sorting (B = 0.746, SE = 0.004, p < 0.001).

These findings suggest that lower rsFC between the CON and DMN is associated with better cognitive performance, potentially serving as a mediating mechanism for the effects of social determinants on cognitive outcomes. Social factors, such as parental education, household structure, and educational opportunities, exert significant direct and indirect influences on cognitive development. Differences by race/ethnicity highlight the role of structural inequities in shaping both neural connectivity and cognitive outcomes.

## 4. Discussion

This study investigated whether CON-DMN rsFC mediates the effects of social determinants, such as educational opportunities and family structure, on cognitive outcomes in youth. Our findings demonstrated that higher educational opportunities and stable family structures were positively associated with better cognitive performance, mediated in part by lower CON-DMN rsFC. These results suggest that social determinants shape neural connectivity patterns, which in turn influence cognitive development during critical formative years.

Our findings align with previous research highlighting the role of social determinants in cognitive outcomes [27–29,50,51]. Educational opportunities, reflected by parental education and neighborhood socioeconomic status, have consistently been associated with better cognitive performance, likely due to enriched environments and greater cognitive stimulation. Similarly, stable family structures provide emotional and logistical support that foster academic success and cognitive growth.

Our findings on the mediating role of neural connectivity, particularly CON-DMN rsFC, add a novel dimension to this literature. Prior studies have established that the CON [14,15,17,52–55] supports various aspects of cognitive function as measured by executive control and goal-directed behavior, while the DMN [56–58] is involved in introspection and self-referential thought. Reduced functional connectivity between these networks is critical for cognitive flexibility and regulation. Consistent with these findings, our results suggest that reduced CON-DMN connectivity supports efficient transitions between introspective and task-focused states, facilitating improved cognitive outcomes in children exposed to positive social determinants.

At least some studies have shown that rsFC can provide a robust framework for examining how social factors influence neural network connectivity and, consequently, cognitive performance. For instance, rsFC between the CON-DMN rsFC has been linked to enhanced cognitive flexibility and executive function in adults [18,36,37]. However, our findings revealed an inverse relationship between cognitive function and CON-DMN rsFC, a result that is not entirely clear and warrants further investigation. One possibility is that the developmental stage may moderate this relationship—while CON-DMN connectivity might support cognitive performance in adults, it could have a different, perhaps even detrimental, impact during earlier developmental periods such as childhood or adolescence. This suggests a complex interplay between neural network maturation and cognitive outcomes, emphasizing the need for additional longitudinal research to better understand these dynamics.

Parental education years showed no significant association with rsFC (B = 0.010, SE = 0.012, p = 0.403). This was a surprising finding given that parental education is often considered a strong indicator of socioeconomic status and is typically linked to positive developmental outcomes. One potential explanation for the null result is that our analysis adjusted for neighborhood educational opportunities, which may capture much of the variability usually associated with parental education. In other words, the local educational environment might play a more critical role in shaping neural connectivity than previously thought, potentially mediating or even overshadowing the effects of individual parental education levels. This finding highlights the importance of considering both individual and community-level factors when examining neurodevelopmental outcomes.

### 4.1. Mechanisms Underlying Observed Relationships

The relationship between social determinants and cognitive outcomes can be partially explained by their influence on neural development [26–31]. Children from families with higher parental education or stable household structures are more likely to experience enriched environments that promote optimal brain development [20–22]. These environmental conditions enhance the development of large-scale neural networks, such as the CON and DMN, reducing excessive connectivity between these networks and improving their functional specialization. Lower CON-DMN rsFC may reflect more efficient neural communication, enabling better regulation of attention and cognitive resources required for academic achievement.

Conversely, children raised in less advantageous social environments may experience chronic stress, fewer cognitive stimuli, and less access to resources, which can disrupt the development and specialization of neural networks. Elevated CON-DMN rsFC in these children may reflect overconnectivity or reduced functional segregation, leading to less efficient cognitive processing and poorer outcomes.

### 4.2. Implications

These findings have implications for understanding socioeconomic disparities in cognitive development. First, they emphasize the importance of improving educational opportunities and supporting family stability as strategies for promoting optimal brain and cognitive development in children. Policies aimed at reducing socioeconomic inequalities, such as access to quality education, parental support programs, and neighborhood investment, may help mitigate the effects of adverse social determinants on neural and cognitive outcomes.

Second, this study highlights the potential of neural biomarkers, such as CON-DMN rsFC, in identifying children at risk for cognitive difficulties due to social disadvantages. Interventions targeting neural connectivity, such as mindfulness-based practices, cognitive training, or neurofeedback, may offer innovative approaches to enhancing cognitive flexibility and executive function, particularly in children from underserved populations.

### 4.3. Limitations

This study has several limitations. First, its cross-sectional design precludes causal inferences regarding the relationships between social determinants, neural connectivity, and cognitive outcomes. Longitudinal data from the ABCD study are needed to establish temporal precedence and causal pathways. Second, while resting-state functional connectivity provides valuable insights, task-based fMRI and structural connectivity measures could offer a more comprehensive understanding of neural mechanisms underlying cognitive regulation. Finally, although the sample was diverse, further research should explore these associations across specific subgroups to identify unique patterns of vulnerability and resilience.

## 5. Conclusion

This study demonstrates that functional connectivity between the CON and DMN partially mediates the effects of social determinants, such as educational opportunities and family structure, on cognitive outcomes in youth. These findings underscore the importance of addressing social inequalities to promote neural and cognitive development and highlight the potential for targeting neural connectivity in interventions aimed at reducing disparities in cognitive outcomes. Future research should build on these findings by employing longitudinal designs and exploring targeted interventions to enhance neural and cognitive resilience in children from disadvantaged backgrounds.

## Figures and Tables

**Table 1.** Summary of Structural Equation Model (SEM)

Independent Variable	Dependent Variable	B	SE	95%	CI	p
**Structural Paths**						
Age	CON-DMN rsFC	−0.067	0.010	−0.086	−0.048	< 0.001
Male	CON-DMN rsFC	0.123	0.010	0.104	0.142	< 0.001
Parent education years	CON-DMN rsFC	0.010	0.012	−0.013	0.033	0.403
Married household	CON-DMN rsFC	−0.024	0.011	−0.045	−0.002	0.034
COI Educational (National)	CON-DMN rsFC	−0.033	0.012	−0.057	−0.009	0.007
Race/ethnicity Black	CON-DMN rsFC	0.031	0.012	0.008	0.055	0.009
Race/ethnicity Latino	CON-DMN rsFC	−0.015	0.011	−0.037	0.007	0.174
Race/ethnicity Asian	CON-DMN rsFC	0.017	0.010	−0.003	0.036	0.100
Race/ethnicity Other	CON-DMN rsFC	0.010	0.010	−0.010	0.030	0.329
Intercept	CON-DMN rsFC	−0.009	0.195	−0.392	0.373	0.963
CON-DMN rsFC	Cognition	−0.035	0.009	−0.053	−0.017	< 0.001
Age	Cognition	0.295	0.005	0.285	0.304	< 0.001
Male	Cognition	−0.035	0.008	−0.051	−0.018	< 0.001
Parent education years	Cognition	0.299	0.010	0.280	0.317	< 0.001
Married household	Cognition	0.078	0.009	0.060	0.097	< 0.001
COI Educational (National)	Cognition	0.081	0.011	0.061	0.102	< 0.001
Race/ethnicity Black	Cognition	−0.139	0.010	−0.159	−0.119	< 0.001
Race/ethnicity Latino	Cognition	0.010	0.010	−0.008	0.029	0.272
Race/ethnicity Asian	Cognition	0.038	0.008	0.022	0.055	< 0.001
Race/ethnicity Other	Cognition	0.003	0.009	−0.014	0.020	0.709
**Measurement**						
Cognition	Reading	0.797	0.003	0.791	0.802	< 0.001
Cognition	Total Composite	0.823	0.004	0.815	0.830	< 0.001
Cognition	Fluid	0.817	0.004	0.808	0.825	< 0.001
Cognition	Picture	0.692	0.004	0.685	0.700	< 0.001
Cognition	Pattern	0.573	0.004	0.565	0.582	< 0.001
Cognition	Card Sort	0.746	0.004	0.738	0.753	< 0.001

rsFC: resting state functional connectivity between the Cingulo-Opercular Network (CON) and Default Mode Network (DMN)
